# Review: chronic and persistent diarrhea with a focus in the returning traveler

**DOI:** 10.1186/s40794-017-0052-2

**Published:** 2017-05-04

**Authors:** Christopher A. Duplessis, Ramiro L. Gutierrez, Chad K. Porter

**Affiliations:** Enteric Disease Department, Infectious Disease Directorate, Naval Medical Research Center, 503 Robert Grant Avenue, Silver Spring, MD 20910 USA

**Keywords:** Travelers’ diarrhea, Chronic diarrhea, Persistent diarrhea, Post-infectious irritable bowel syndrome, GeoSentinel surveillance, Enteropathogens, Giardiasis

## Abstract

**Background:**

Travelers’ diarrhea is a common malady afflicting up to 50% of travelers after a 2-week travel period. An appreciable percentage of these cases will become persistent or chronic. We summarized the published literature reporting persistent/chronic diarrhea in travelers elucidating current understanding of disease incidence, etiology and regional variability.

**Methods:**

We searched electronic databases (Medline, Embase, and Cochrane database of clinical trials) from 1990 to 2015 using the following terms: “chronic or persistent diarrh* and (returning) travel* or enteropathogen, GeoSentinel, and travel-associated infection. Included studies published in the English language on adult returning travelers (duration < 3-months) reporting denominator data. Point estimates and standard 95% confidence intervals were calculated for incidence using a random-effects model. Study incidence heterogeneity rates were assessed using *x* ^2^ heterogeneity statistics, graphically represented with Forest plots.

**Results:**

We identified 19 studies meeting the inclusion criteria (all published after 1999). 18 studies reported upon the incidence of persistent/chronic diarrhea as a syndromic diagnosis in returning travelers; one study reported adequate denominator data from which to assess pathogen specific etiology. *Giardiasis* comprise an appreicaible percentage of infectious mediated persistent/chronic diarrhea in returning travelers. The overall estimate of persistent/chronic diarrhea incidence was 6% (0.05–0.07) in 321,454, travelers; with significant heterogeniety observed across regions. The total number of regional travelers, and point estimates for incidence (95% CI) for Latin American, African, and Asian travelers were [15816 (0.09 [0.07–0.11]), 42290 (0.06 [0.05–0.07]), and 27433 (0.07 [0.06–0.09])] respectively. We identified lower published rates of chronic diarrhea from Sub-Saharan Africa relative to [North Africa, South Central Asia, and Central America]. Persistent/chronic diarrhea ranked fourth as a syndromic diagnosis in all regions.

**Conclusions:**

Persistent/Chronic diarrhea is a leading syndromic diagnosis in returning travelers across all regions. The 6% incidence [proportionate morbidity (PM) of 60] observed in over >300,000 global travelers is comparable to prior estimates. We identified lower published rates of chronic diarrhea from Sub-Saharan Africa relative to [North Africa, South Central Asia, and Central America]. *Giardiasis* comprises an appreciabile percentatge of travel-associated infectious mediated persistent/chronic diarrhea. There’s a dearth of published data characterizing the incidence of specific enteropathogenic etiologies for persistent/chronic diarrhea in returning travelers.

## Background

Almost one billion individuals traveled internationally in 2011 [1]. Travelers’ diarrhea (TD) is the most common malady afflicting travelers, and several observational studies report an incidence of 50% after a 2-week travel period [2, 3]. High risk areas for experiencing travelers diarrhea according to the CDC include most of the developing areas of the world, particularly within the tropical and subtropical areas including SubSaharan Africa (excluding South Africa), SouthEast Asia, SouthCentral Asia, the Middle East, Latin America (South and Central America excluding Chile and Argentina) and Oceana [2, 4, 5]. Intermediate risk regions include the Caribbean nations, South Africa, Argentina, Chile, Eastern Europe, Russia, China and Portugal [2, 4, 5]. Bacteria account for up to 90% of identified infectious etiologies for acute TD, predominately enterotoxigenic *Escherichia coli* (ETEC), and enteroaggregative *E. coli* (EAEC), although there is regional variability [5, 6]. Of public health importance, travelers are recognized as an important vector for transmission of emerging and multi drug resistant (MDR) enteropathogens globally, mandating global public health surveillance [2].

An estimated 3–10% of travelers experience persistent diarrhea (diarrhea exceeding 2-weeks) [7] while upwards 4% of returning travelers experience chronic diarrhea (diarrheal exceeding 4-weeks) [5–9]. These estimates vary widely dependent on geographical location, travel duration, itinerary, population, and preceding utilization of pre-travel clinic education and counseling. As our research failed to identify the incidence of specific enteropathogenic etiologies of persistent or chronic diarrhea in returning travelers, and noted that most syndromic diagnoses were biased towards the definition of chronic diarrhea, we will consolidate both definitions as persistent/chronic diarrhea.

We propose to catalogue four categories of persistent/chronic diarrhea in the returning traveler (as referenced in [5, 7, 9, 10] as follows: 1) infectious [presumably biased towards parasitic [5] (although a host of bacterial enteropathogens contribute to the burden of chronic infectious diarrhea)] [7, 10–12]; 2) presumed infectious (tropical sprue, Brainerd’s diarrhea); 3) post-infectious sequelae [post infectious irritable bowel syndrome (PI-IBS), lactose intolerance, small intestinal bowel obstruction (SIBO)]; and 4) unmasked (presumably via an infectious disease exposure/trigger) [bile acid malabsorption (BAM), inflammatory bowel disease (IBD), celiac disease, microscopic colitis]—see Table 1. The terminology “unmasked” suggests that an infectious exposure may be a trigger in a cascasde of events which induces disease in a susceptible population.

**Table 1:** Categorization and brief exposition of known etiologies for persistent/chronic diarrhea in returning travelers

1). Infectious	Bacterial: [5, 9, 10] *Campylobacter* spp., [5, 9, 10] *Shigella* spp., [5, 9] *Aeromonas* spp., [5, 9, 10]*Clostridium difficile*, [5, 9, 10] *Salmonella* spp.
	Parasitic: [5, 7, 10] *Cryptosporidium parvum*, [5, 7, 9, 10] *Entamoeba. histolytica*, [5, 7, 9, 10] *Giardia lamblia*, [5, 9, 10] *Isospora belli*, [5, 7, 10] *Cyclospora cayetanensis*, [10] *Microsporidia spp*., [7, 9] *Dientameba fragilis*
	Helminthic: [5, 9] *Strongyloides stercoralis*,, *and* [9]*Schistosoma* spp.
2). Presumed Infectious	[9] Brainerd’s Diarrhea, [7, 10] Tropical Sprue
3). Post infectious sequelae	[5, 9, 10] Lactose intolerance, [5, 7, 9, 10] Post infectious irritable bowel syndrome (PI-IBS), [7, 9, 10] Small intestinal bowel overgrowth (SIBO)
4). Unmasked	[5, 7, 10] Celiac disease, [5, 7, 9, 10] Inflammatory bowel disease (IBD), [10] Microscopic colitis
[5] deSaussure, 2009; [7] Connor, 2005, [9] DuPont, 1996, [10] Gutierrez, 2012	
Brief Exposition of Etiologies for Persistent/Chronic Diarrhea in Returning Travelers	
1). Infectious	Bacterial
	1). Aeromonas is a gram negative bacilli, oxidase positive and associated with freshwater identified in chronic watery diarrhea cases and associated with asymptomatic carriage [11]. Diagnosis requires stool culture and treatment ensues with a fluoroquinolone or a third-generation cephalosporin. 2). *Campylobacter spp*. (primarily *C. jejuni*) is a major cause of acute diarrhea globally. It has been implicated in chronic diarrhea in travelers. *Campylobacter spp*. are foodborne illnesses, which may be transmitted person-to-person or via exposures to animal vectors (poultry). It may produce watery to bloody diarrhea (dysentery) and lead to post-infectious complications including reactive arthritis, PI-IBS, and Guillain-Barre syndrome. It is diagnosed by stool culture, and treated with macrolide or fluoroquinolone antibiotics (acknowledging increasing resistance to the latter in SE Asia) [10]. 3). *Clostridium difficile*, a gram-positive anaerobic bacillus and the cause of pseudomembranous colitis, has emerged as a major infectious etiology of both acute and diarrhea (watery to bloody diarrhea), often associated with profound leukocytosis, and chronic diarrhea in travelers, regardless of antibiotic exposure [12]. 4). Shigella spp. are aerobic gram-negative rods (non-motile, non-spore forming) in the family Enterobacteriaceae partitioned into four groups (group A: S. dysenteriae; Group B, S. flexneri; Group C, S. boydii; Group D, S sonnei). Shigella spp. is s a major cause of travelers associated acute diarrhea, endemic and travelers assoiated dysentery, and chronic diarrhea in travelers. It is transmitted by contaminated food and water or fecal orally. It may produce watery or frankly bloody diarrhea. Post infectious complications include reactive arthritis, and PI-IBS. It is diagnosed by stool culture and treated via macrolide or fluoroquinolone antibotics, although mirroring assertions for all bacterial enteropathogens, resistance is increasing [10]. 5). Salmonella (non-Typhi) is a foodborne illness which may also be transmitted by aimals (reptiles, birds). It may produce watery diarrhea and occasionally may be culpable in producing frank dysentery and bacteremia. Post infectious complications include reactive arthritis and PI-IBS. It is diagnosed by stool culture. Treatment is withheld except for severe symptoms, or in elderly or very young patients [10].
	Parasitic
	1). Giardiasis is a ubiquitous parasitic infection exhibiting global transmission. This infection produces the classic steatorhea emanating from malabsorption associated with bloating, nausea, and emesis often mimicking IBS. The trophozoites localize in the small intestine. Giardia may be transmitted via contaminated food, water or direct person-person contact (fecal oral spread common in day care centers, residential institutions, and among MSM). The diagnosis is best rendered with a stool ELISA measuring the Giardia antigen often coupled with the *Cryptosporidium parvum and Entameba histolytica*. Treatment is effected with a 7–10 days course of metronidazole [10]. 2). *Cryptosporidium parvum* is a coccidian protozoa with fastidious oocysts which typically causes chronic watery diarrhea (often associated in outbreaks) in immunesuppressed individuals, but has been identified as a cause of persistent/chronic diarrhea in travlers [6]. The oocytes resists chlorination, is transmitted fecal orally, linked to poor sanitation, diagnosed via stool microscopy (O&P and partial acid fast staining) or antigen testing (ELISA) [32]. 3). *Cyclospora cayetanensis* is a coccidian protozoan (humans the only reservoir) producing a more severe clinical spectrum of diarheal disease than that of *Cryptosporidium parvum*. Diarrheal outbreaks have been reported in Peru, Nepal and Guatemala (imported Guatemalan raspberries) in the U.S. [6]. In contradistinction *to Cryptosporidium spp*., Cyclospora requires sporulation in the environment, thus human-human transmission is unlikely [6, 32]. The diagnosis requires partial acid-fast staining of a stool specimen (noting cysts are 10 μm in size (vice 5 μm for *Cryptosporidium* and 20–30 μm for *Isospora belli*-described below)) and responds to treatment with trimethoprim-sulfamethoxazole. 4). *Isospora belli* is a large protozoan responsible for diarrhea in immunocompromised patients and identified in persistent/chronic diarrhea in returning travelers. It is another ubiquitous low virulence organism markedly underdiagnosed. Diagnosis and treatment is similar to *Cyclospora* [6]. 5). *Microsporidia* are intracellular spore forming protozoans referring to *Enterocytozoon bieneusi* and *Encephalitozoon intestinalis* genotypes associated with human intestinal infections [6]. This is another ubiquitous organism culpable for diarrhea in immune-compromised patients, and persistent/chronic diarrhea in returning travelers, and is likely underdiagnosed given difficulty in diagnosis (improved exloiting PCR methods) [33]. Unlike the *Cryptosporidia*, *Cyclospora spp*., *and Isospora spp*. diagnosed via partial acid fast staining, Microsporidia requires modified Trichrome staining [6]. Treatment options include albendazole. 6). Amebiasis, due to *Entamoeba histolytica* can present with a spectrum of disease manifestations including acute and persistent/chronic diarrhea in retruning travelers. This protozoan can be invasive (producing flask shaped abscesses) and may produce severe abdominal pain, fever, and bloody stools (dysentery). Complications include formation of liver abscesses. Transmission occurs via contaminated food and water, and fecal-oral contact. Diagnosis occurs via microscopy on stool specimens, stool antigen ELISA or PCR [5, 32]. Treatment entails metronidazole for invasive disease coupled to paromomycin or diloxanide as a luminal agent (to eradicate remaining cysts).
	1). *Strongyloides stercoralis* is a nematode infection, primarily identified in tropical and sub-tropical areas globally, transmitted by exposure to contaminated soil. Filariform larvae penetrate the skin entering the lymphatics and migrating to the lung, thence the small intestines. The infection may remain dormant for decades, symptomatic after immune-suppression (steroids). It may also present with chronic diarrhea upon exposure during travel. The diagnosis ensues with culture, microscopy (identifying the larvae), and serology. Treatment is best effected with Ivermectin repeated at 2-weeks to treat the autoinfective cycle [34]. 2). *Schistosoma spp*.: The etiology of schistosomiasis (bilharzia), these are blood flukes, (helminthic parasites, in the class Trematoda). They are prealent throughout the tropics (particularly in SSA and ME). Schistosomiasis involves a complex life cycle with water mollusks serving as intermediate hosts, infective cercariae entering the skin, honing to species specific trophic organism (bladder for S. hematobium, portal venous system for S mansoni), release of eggs and becoming miracidium once deposited in the environment to reinfect mollusks. Diagnosis requires stool, urine, or tissue evaluation for eggs, and serologic assays. Treatment with praziquantel quite effective requiring repeated dosing to prevent relapses [35].
2). Presumed Infectious	1). Brainerd’s diarrhea is an epidemic form of watery secretory diarrhea. It was first described after an outbreak which emerged in Brainerd Minnesota [36]. The symptoms exhibit a median duration of 15–16.5 months and may last for several years. Histopathologic analysis of colonic biopsies revieal patchy lymphocytic colonic inflammation. Although an infectious trigger is presumed, no microbiological entity has been identified to date. Risk factors have been identified via epidemiological investigations and include consumption of contaminated water and unpasteurized milk. Secondary transmission is rare [36]. 2). Tropical sprue is a condition of unknown etiology characterized by chronic diarrhea, weight loss, fatigue, steatorrhea, and malabsorption, usually associated with evidence of micronutrient deficiency (anemia, folate, and vitamin D deficiency). It is endemic in tropical regions, and rare in North America and Europe [37]. It is thought to be precipitated by an unknown infectious trigger [38]. Tropical sprue represents one of the most common chronic enteropathies of the developing world mirroring the most common chronic enteropathy (celiac sprue) of the developed world, manifesting similar histologic features. This enteropathy is multifactorial characterized by bacterial overgrowth (SIBO), deranged gut motility, parasitic infections, and hormonal and histopathologic abnormalities [39]. Histopathologic analyses are similar to that noted in celiac disease exhibiting villous atrophy, crypt hyperplasia, and epithelial lymphocytosis [40]. Diagnosis requires excluding alternative etiologies (celiac disease, lactose intolerance, SIBO, IBS) and treatment is effective with empiric antiotics and folate supplementation [41].
3). Post infectious sequelae	1). Lactose Intolerance: Gastroenteritis may precipitate secondary enzymatic disaccharidase deficiency. These enzymes are located within the microvilli [brush border] of small intestinal enterocytes, which are responsible for carbohydrate (lactose, sucrose) hydrolysis contributing to malabsorption (maldigestion) and diarrhea. Perhaps the quintissential mucosal malabsorption syndrome, lactose intolerance involves a deficiency in the enzyme lactase which is responsible for lactose hydrolysis. The unabsorbed lactose leads to mild osmotic diarrhea associated with abdominal pain, cramping, bloating, and diarrhea. The enzymatic deficiency is typically transient, but in a minority (genetically predisposed) may be permanent [7]. Predisposed patients may harbor minimal digestive or absorptional reserve compensated until the superimposed enteritis depletes the remaining brush-border enzymatic function [38]. The contemporary diagnosis is primarily clinical [soliciting a history of diarrhea associated with lactose consumption]. Confirmatory testing includes identifying a low stool pH < 6 (a sensitive screening assay), accompanied by histopathologic evaluation with/without a mucosal lactase assay from a mucosal biopsy retrieved during endoscopy and/or hydrogen breath testing [7, 38]. 2). PI-IBS: Mounting evidence supports an association between enteric infection, including TD, and IBS (hence referred to as post-infectious IBS (PI-IBS)) [30, 42]. PI-IBS has been associated with all families of enteropathogens [5]. Two meta-analyses have demonstrated a 6- to 7-fold increase in the risk of developing IBS following an antecedent episode of gastroenteritis [43, 44]. Recent data culled from the GeoSentinel network (1997–2011) quote a PI-IBS incidence of 2–5% [19, 29] and the prevailing consensus is that the incidence likely resides within a window of 5–10% [30, 31]. PI-IBS has been associated with increased duration and severity of the antecedent GI illness, comorbidities (anxiety, depression), concomitant psychological stressors, and enteropathogenic virulence and invasiveness (stronger associations with *Yersinia spp*, *Campylobacter* > *Shigella* > *Salmonella*) [5, 7, 43–46]. 3). Small intestinal bowel overgrowth (SIBO): The small bowel is typically minimally colonized by bacteria (possessing < 10^4^ organisms/mL) (unlike the large bowel possessing upwards 10^12^ cfu/ml) due to gastric acidity, intestinal peristalsis and a competent ileocecal valve. SIBO may be an isolated finding, or coexist with multiple GI derangements [dysfunctional intestinal motility, mucosal pathologies, reduced gastric acid barrier or dysfunctional anatomy stemming from surgery (blind loops emanating from gastric bypass)] or evolve from intestinal stasis induced by an episode of gastroenteritis and perpetuated by immune-deficiencies [7, 47]. The gold standard for diagnosing SIBO encompasses a quantitative culture of an aspirate of luminal fluid (via upper endoscopy) eclipsing 10^5^–10^6^ [normal < 10^4^] organisms/mL or by exploiting breath testing. Empiric antibiotic trials (including anaerobic coverage) may be both diagnostic and therapeutic (i.e., the non-absorbable antibiotic Rifaximin [47].
4). Unmasked	1). Celiac disease: celiac disease is an immune-based reaction to dietary gluten (storage protein for wheat, barley, and rye) that primarily affects the small intestine in those with a genetic predisposition and resolves with exclusion of gluten from the diet. Celiac disease is the most common small bowel inflammatory enteropathy in the Western world (afflicting 1% of Caucasians) and presents with diarrhea, steatorrhea, weight loss, bloating, flatulence, post-prandial abdominal pain, and a host of extraintestinal complications attributed to malabsorption including osteoporosis, neurologic and skin disorders (dermatitis herpetiformis) [47]. Its prevalence is increasing (attributed in part to detection bias) yet likely remains under-diagnosed [48]. The prevalence is higher in those manifesting autoimmune diseases [insulin dependent diabetes, thyroid disease, or primary biliary cirrhosis]. Celiac disease is classified as a malabsorptive disease associated with watery diarrhea mimicking IBS-D [48]. The differential diagnosis includes microscopic colitis, SIBO, tropical sprue, autoimmune enteropathy, hypogammaglobulinemic sprue, Whipples’ disease, Crohn’s disease, eosinophilic enteritis, intestinal lymphoma, TB, graft-host disease, and pancreatic exocrine insufficiency [48]. Laboratory abnormalities include [abnormal liver function tests, iron deficiency anemia, and micronutrient deficiencies (folic acid, vitamin B12, vitamin D, zinc, copper, fat soluble vitamins)]. The diagnosis is predicated upon serologic testing of celiac-specific antibodies with confirmation by duodenal mucosal biopsies revealing villous injury (effacement). 2). Inflammatory Bowel Disease: IBD encompasses ulcerative colitis (UC) and Crohn’s disease. IBD primarily affects patients in a bimodal age distribution with the majority of cases arising between the ages of 15 and 40 years. However, it may present in younger and older individuals. The disease involves exacerbations or flares manifesting with a spectrum of symptoms which may encompass abdominal pain, weight loss, diarrhea (with or without blood, and mucus), and frank hematochezia. Extraintestinal symptoms are prevalent including ocular (uveitis, episcleritis), musculoskeletal (arthritis, back pain), and/or dermatologic (pyoderma gangrenosum (UC); erythema nodosum). Triggers for IBD are unknown but the disease is multifaceted involving the interactions in host genes, immunity, and environment [7]. Interestingly, recent emerging research implicated an increased risk of IBD following acute infectious gastroenteritis (IGE) (OR 1.53, 95% CI 1.4–1.7) after controlling for important covariates including prior IBS diagnosis [49]. 3). Microscopic colitis: microscopic colitis is an inflammatory bowel disease (Ohlsson, [50]) which mirroring bile acid malabsorption (BAM), is increasingly recognized as a common cause of chronic watery secretory diarrhea, (manifesting nocturnally as opposed to IBS) exhibiting increasing incidence [10–20% of chronic diarrhea cases; reaching 30% of attributable cases of chronic water diarrhea in the elderly (>65)] [48, 51]. The increased incidence may be attributed in part to detection bias (increased recognition and increased colonoscopic evaluation incorporating mucosal biopsies potentiating histopatholgic evaluation) [51]. It encompasses two primary diseases based upon histopathology, collagenous colitis (CC) and lymphocytic colitis (LC). This disease should always be considered in older patients with persistent nocturnal diarrhea unresponsive to fasting, and in the differential of diarrheal predominant IBS [50, 51].
[6] Goodgame, (2003); [11] Vila (2003); [12] Neuberger (2013); [32] Okhuysen (2001); [33] Wichro, 2005; [34] Montes, 2010; [35] Clerix, 2011; [36] Mintz, 2003; [37] Batheja, 2010; [39] Nath, 2005; [41] Farthing, 2002; [38] Landzberg, 2005; [40] Langenberg, 2014; [42] Kennedy, 2014; [30] Dupont, 2014; [43] Halvorson, 2006; [44] Thabane, 2007; [19] Mendelson, 2014; [29] Harvey, 2013; [31] Alajbegovic, 2012; [45] Hong, 2014; [46] Porter, 2013; [47] Murray 2012; [48] Sandhu, 2012; [49] Porter, 2008; [50] Ohlsson, 2015; [52] Ingle, 2014; [51] Yen, 2011	

Our current understanding of the etiology of persistent/chronic infectious-mediated diarrhea in the returning traveler is limited to case studies, case series, and cross-sectional studies. Given the large number of international travelers and the ever increasing geographical destinations, persistent/chronic diarrhea is likely to increase as a public health threat. Epidemiological data on the infectious etiology of persistent/chronic diarrhea are needed to develop evidence-based guidelines for disease management. Therefore, we conducted a systematic review of the published literature to summarize the current data on the incidence, etiology and regional variability of persistent/chronic diarrhea among returning travelers.

## Methods

### Search strategy

We conducted a search of electronic databases (Medline, Embase, and the Cochrane database of clinical trials) from 1990 to 2015, with the following terms: “chronic or persistent diarrh* and (returning) travel* [allowing for travel, traveler, and variable spelling (diarrhea or diarrhoea) and (traveler or traveller)]; GeoSentinel Surveillance and diarrh*; Geosentinel (based on review of references from the aforementioned search keywords Geosentinel Surveillance and diarrh*), chronic or persistent diarrh* and enteropathogen, and travel-associated infection (predicated upon idetnifying potential articles within the references of all aforementioned search keywords).

### Inclusion/Exclusion Criteria

All articles were reviewed for eligibility criteria. To be included, studies were required to 1) report on adult (≥18 years) travelers presenting for travel-related illness at a health-care facility (excluding survey based data), 2) be published in the English language from 1990 to 2015, 3) report denominator data (extractable incidence rates) of persistent and/or chronic diarrhea among returning travel populations (experiencing travel duration for up to 3 months).

### Data abstraction

The following data were abstracted and entered into a MS Excel® worksheet for analysis: author, publication year, study years, travel origination, population demographics, travel destination, number of travelers, PM, diarrheal etiology, and burden of persistent/chronic diarrheal disease relative to all cause travel related morbidity.

### Analysis

Incidence rates and standard 95% confidence intervals (for all travelers (global) and region specific [Latin America, Africa and Asisa]) were estimated using a random-effects model (DerSimonian & Laird) [13]. Heterogeneity in study incidence rates was assessed using a *χ* ^2^ statistic, and graphically represented with Forest plots. Statistical analyses were performed with Stata Version 10 (StataCorp., College Station, TX).

## Results

**Fig. 1:**
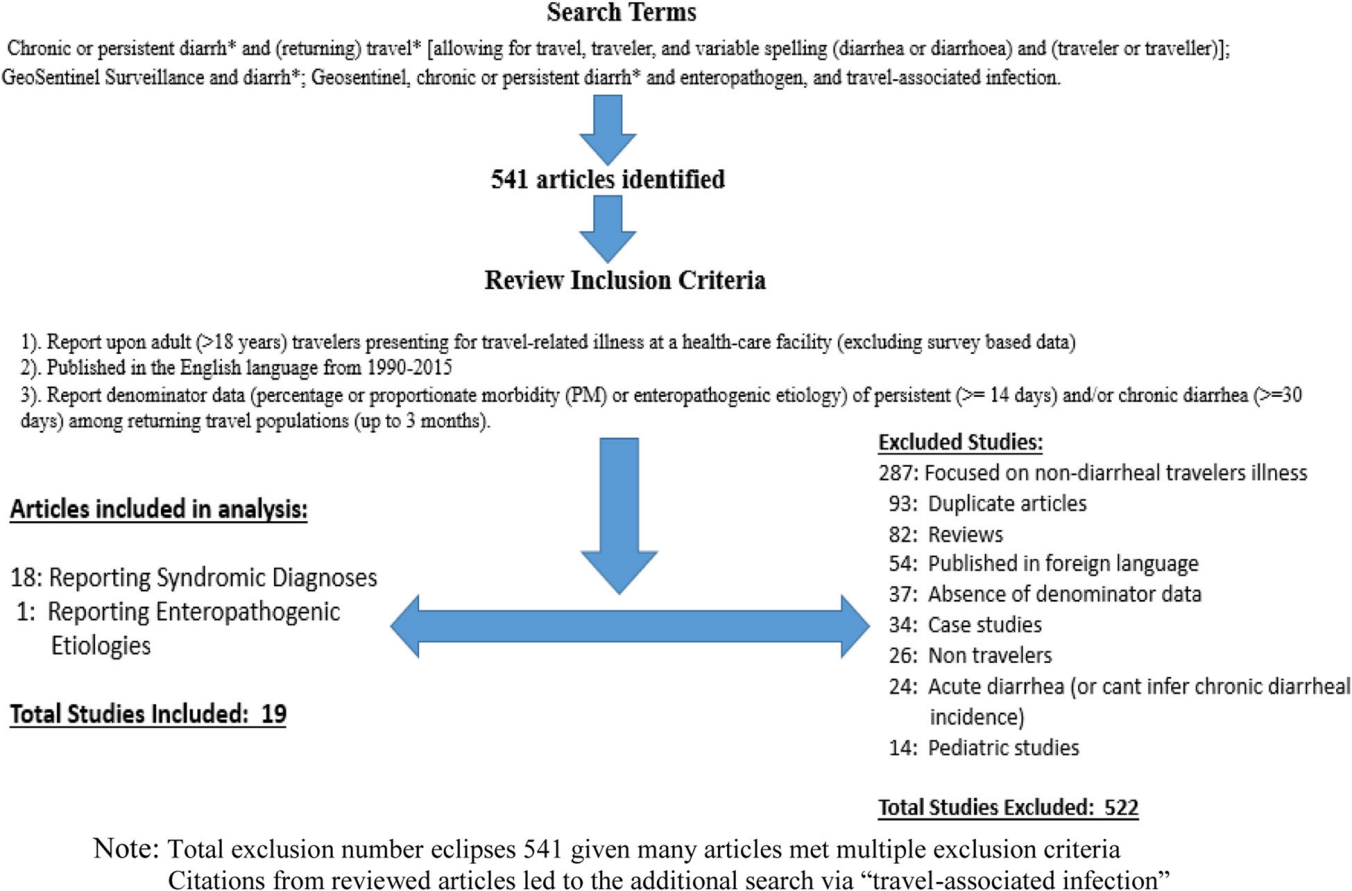
Flow Chart Depicting Search Methodology

Our initial search resulted in 541 articles from which we identified 19 studies meeting the inclusion criteria. Of those, 18 reported on the incidence of persistent/chronic diarrhea as a syndromic diagnosis in returning travelers while one study reported adequate denominator data to enable estimates of pathogen-specific etiology (Table 2). The majority (287; 53%) of articles were excluded due to reporting on non-diarrheal travel related illness. Additional exclusionary criteria included duplicate articles (93; 17.2%), reviews (82; 15.2%), publication in foreign language (54; 9.9%), failure to provide denominator data (37; 6.8%), case studies (34; 6.3%), non-travel study populations (26; 4.8%), and pediatric populations (14; 2.6%) (Fig. 1). Odolini et al. reported data from two separate years of surveillance and data for each year were entered as separate observations [14]. Similarly, Gautret et al. [15] reported on separate cohorts stratified by age (young and elder cohorts). These data were extracted and entered as separate observations yielding a total of 20 observations for syndromic diagnoses [15]. The predominant reasons for failing eligibility in identifying enteropathogenic etiologies for persistent/chronic diarrhea in returning travelers were a lack of pathogen specific incidence reporting, and failing to partition acute and chronic diarrheal presentations.

**Table 2:** Studies identifying the syndromic diagnosis of chronic diarrhea with ranking relative to all assessed syndromic diagnoses^a^

Author	Publication Year	Surveillance Years	Origin of Travelers	%Male	Age Median (IQR/Range) Mean (std)	Destination^c^	Number of Travelers	PM^d^ Chronic Diarrhea (CD)	Rank Among Syndromic Diagnoses	Leading Syndromic Dignoses [Rank & PM]^b^
Freedman	2006	1996–2004	Global	52	33 (26–45) IQR	Global	17353	113	4	#1: AFI 226 #2: AD 222 #3: D 170
				46	37 (27–50) IQR	Carribean	1115	132	4	#1: D: 261 #2: AD 196 #3: AFI 166
				55	30 (23–42) IQR	South America	1675	130	4	#1: D 264 #2: AD 219 #3: AFI 143
				48	32 (24–45) IQR	Central Am/Mexico	1326	173	3	#1: AD 234 #2: D: 225
				56	34 (27–45) IQR	SSA	4524	57	7	#1: AFI 371 #2: AD 167 #3: D 127
				50	32 (25–45) IQR	SC Asia	2403	129	4	#1: AD 327 #2: AFI 171 #3: D 130
				53	32 (25–42) IQR	SE Asis	2793	97	4	#1: AD 248 #2: D 212 #3: AD 210
Leder	2006	1996–2004	Global	49	36.4 (1–85)	SSA	2062	40	4	#1: AFI 180 #2: AD 130 #3: D 130
				48	34.8 (1–91)	Asia	4615	60	5	#1: AD 210 #2: D 140 #3: AFI 120 #4: R 60
				47	35 (1–83)	Latin America	2581	80	4	#1: D 230 #2: AD 150 #3: AFI 100
Greenwood	2008	2000–2005	Global	--	--	Global	22569	57	--	--
Davis	2008	1998–2007	Global	--	--	SE Asia	6913	66	6	#1: D 209 #2: AFI 205 #3: AD 168 #4: ND GI 76 #5: R 73
				--	--	SC Asia	3543	116	3	#1: AD 294 #2: AFI 168
Chen^e^	2009	1996–2008	Global-Short Term^e^	50	38 (NA)	Global	24807	45	4	#1 AD 123 #2: D 60 #3: GI Parasites 55
			Global-Long Term^e^	57	33 (NA)	Global	4039	50	1	#1: CD 50 #2: G 36 #3: IBS 36
				---	---	South America	----	58	3	#1: CL: 72 #2: IBS: 66
				---	---	Central Am/Mexico	----	108	1	#1: CD 108 #2: IBS 97 #3: AD 37
				---	---	North Africa	----	--	--	#1: G 44 #2: AD 44 #3: IBS 37
				---	---	Middle East	----	31	2	#1: G 109 #2: CD 31
				---	---	SC Asia	----	87	1	#1: CD 87 #2: G 86
				---	---	SE Asia	----	54	2	#1: D 63
Mendelson	2010	1997–2009	Global	52		SSA	13460	56	6	#1: AFI 314 #2: AD 166 #3: D 118 #4: ND GI 91 #5: R 64
				50	--	South Africa	823	66	5	#1: AFI 390 #2: D 156 #3: AD 130 #4: R 68
Field	2010	2008	European	51.1	36 (0–89)	Global	6957	70	5	#1: AFI 200 #2: AD 170 #3: D 120 #4: R: 80
				---	---	Carribean	116	80	4	#1: D: 300 #2: AD 240 #3: AFI 200
				---	---	South Am	477	90	4	#1: D: 180 #2: AD: 150 #3: AFI 150
				---	---	Central Am/Mexico	133	110	5	#1: AD 320 #2: D: 260 #3: AFI 160
				---	---	North Africa	366	160	2	#1: AD 360
				---	---	SSA	1832	50	6	#1: AFI 330 #2: AD 180 #3: D 120
				---	---	Middle East	109	90	5	#1: AD 310 #2: AFI 150 #3: D 130
				---	---	SC Asia	683	110	3	#1: AD 390 #2: AFI 170
				---	---	SE Asia	637	90	5	#1: AFI 250 #2: D 220 #3: AD 210 #4: R 100
Schlagenhauf	2010	1997–2007	Global (Males)	100%	35.9 (NA)	Global	29265	53	4	#1 AD 216 #2 AFI 174 #3 R 107
			Global (Females)	100%	34.4 (NA)	Global	29643	65	4	#1 AD 246 #2 R 116 #3 AFI 113
Flores-Figueroa	2011	1996–2010	NA Europe	46	35.9 +/− 14.7	Central America	4779	114	4	#1:AD 235 #2: D 201 #3: AFI 128
Gautret	2012	1997–2009	Global Elderly >60	49	65 (60–98)	Global	7034	59	7	#1: AD 167 #2: R 146 #3: D 145 #4: AFI 126 #5: ND GI 76 #6: MSK 65
						Latin America	1161	102	5	#1: D 188 #2: AD 152 #3: AFI 121
						Africa	1543	70	5	#1: AFI 225 #2: AD 150 #3: D 148 #4: R 79
						Middle East	179	78	6	#1: D 179 #2: AD 168 #3: R 156 #4: AFI 106
						Asia	3311	37	9	#1: AD 197 #2: R 185 #3: D 127
Gautret	2012	1997–2009	Global 18–45	55	30 (18–45)	Global	56042	65	5	#1: AD 229 #2: AFI 183 #3: R 103 #4 D 133
Odolini	2012	2008	Europe	51	36 (27–48) IQR	Global	6957	40	4	#1: AD 60 #2: Viral 60 #3: Malaria 50
				---	---	North Africa	330	80 (140)	2	#1: AD 160 #2: CD 80 #3: PI-IBS 60
				---	---	SC Asia	676	60	4	#1: G 110 #2: AD 110
				---	---	SE Asia	631	50	4	#1: Dengue 100 #2: Viral: 100 #3: AD: 90
				---	---	South America	351	50	3	#1: AD: 70 #2: Viral 70
Odolini	2012	20092009	Europe	50	35 (27–47) IQR	Global	6392	40	4	#1: AD 60 #2: Malaria 60 #3: Viral 60
				---	---	North Africa	248	60 (150)	3	#1: AD 150 #2: CD 60 #3: PI-IBS 70
				---	---	SC Asia	550	70	4	#1: G 110 #2: AD 90 #3: Campy 70
				---	---	SE Asia	678	40	5	#1: Dengue 100 #2: AD 80 #3: Viral #4: Hookworm 60
				---	---	South America	374	50	4	#1: AD: 80 #2: Malaria 60 #3: Hookworm 50
Harvey^f^	2013	1997–2011	U.S.	49	34 (med)	Global	13,059	80	5	#1: Acute Diarrhea 220 #2: GI Other 150 #3 AFI 140 #4 D 120 #5: CD 80
Mendelson^g^	2014	1997–2011	NA Europe	52	35 (0–92)	Africa	16893	50 (70)	4	#1 Malaria 130 #2: Acute diarrhea 80 #3: Viral Syndrome 70 PI-IBS 20
				---	37 (0–87)	North Africa	2474	110 (150)	2	#1: AD 230 #2: CD 110 #3: PIIBS 40
				---	35–37 (0–92)	Central/West Africa	7446	30	6	#1: Malaria 250 #2: AD 100 #3: Viral 70 #4: G 40 #5: AFI 30
				---	34 (0–89)	East Africa	5516	50 (70)	4	#1: AD 120 #2: Viral 80 #3: Malaria 50 #4: CD 50 #5: PI-IBS 20
				---	40 (0–89)	South Africa	1457	50	4	#1: R 190 #2: Viral 120 #3: AD 70
Boggild	2014	2009–2011	Canadian	42	38 (0–89)	Global	2010	82 (166)	3	#1: AD 85 #2: PI-IBS 84
Wilson	2014	1997–2013	Global	55	33 (0–78)	Brazil (South Ameica)	1586	44	4	#1: D 397 #2: AFI 187 #3: AD 92
Monge-Maillo	2014	1989–2010	Spain	44	36 (28–44)	SSA	209	10	--	----
				35		Latin America	142	63	--	--
Hagmann	2014	2000–2012	U.S.	48.6	33 (0–94)	Global	9624	105	6	#1: AD-- #2: AFI 182 #3: GI other 178 #4: Dem 166 #5: Respiratory 108
				---	---	Caribbean	---	---	---	#1: AD: 156 #2: Dengue 69 #3: Viral 50 #4: R 39 #6: PIIBS 36
				---	---	South America	---	34 (121)	5	#1: AD 197 #2: PIIBS 87
				---	---	Central Am/Mexico	---	30 (99)	4	#1: AD 275 #2: PIIBS 69 #3: D 46
				---	---	North Africa	---	40 (103)	4	#1: AD: 280 #2: Giardia 85 #3: PIIBS: 63
				---	---	SSA	---	(28)	7	#1: Malaria 127 #2: AD 145 #3: Giardia 42 PIIBS 28 #7
				---	---	Middle East	---	(57)	4	#1: AD: 220 #2: Shistosomiasis 68 #3: G 57 #4: PIIBS 57
				---	---	SC Asia	---	28 (83)	3	#1: AD: 250 #2: Giadia 81 #3: PIIBS: 55 #4: R: 36 CD: 28 #8
				---	---	SE Asia	---	31 (84)	5	#1: AD 194 #2: PIIBS 53 #3: Dengue 41 #4: R 32
Schlagenhauf^h^	2015	2008–2012	European	51	35 (27–48)	Global	32136	40	4	#1: Malaria 70 #2: AD 70 #3: Viral 55 PI-IBS 20

^a^There were 18 total reviews identified. Two studies reported upon two discrete cohorts, therefore 20 total reviews are reported. Most reviews reported relative ranking of the syndromic diagnoses and these are delineated with leading syndromic diagnoses

^b^Syndromic Diagnoses: CD: Chronic Diarrhea; AD Acute Diarrhea; G: Giardia; IBS: Irritable bowel disease; D: Dermatologic; CL: cutaneous leishmaniasis; AFI: Acute febrile illness; R: respiratory disease; ND GI: Non diarrheal GI disease: MSK: musculoskeletal

^c^SSA: Subsaharan Africa; Central Am: Central America; SC Asia: South Central Asia; SE Asia: South East Asia

^d^PM: proportionate morbidity—the number of cases per 1000 travelers surveyed

^e^Including study by Chen e al which reported upon both Long Term Travelers defined as > 6-months; Short Term Travelers < 1-month

^f^5% of enrollees were <18 years old

^g^7% of enrollees were < 19 years old

^h^Approximate values culled from graphs

We identified a single study reporting upon the pathogen-specific etiology for persistent/chronic diarrhea in the returning traveler, delineating transparent denominator data enabling estimates of incidence rates [16]. A total of 116 consecutive patients experiencing persistent/chronic diarrhea post-travel were enrolled at two clinics between 1995 and 1996 (Netherlands and Belgium). *Giardia* was the most common infection in 16.4% of specimens followed by *Campylobacter* (*6.1*%); *Shigella* (*3.5*%); *Cyclospora* (*3.5*%); *Salmonella spp*. (*0.9*%); *and Entamoeba histolytica* (*0.9*%). Paschke et al. [17] conducted a similar study, but did not stratify the enterpoathogens across the acute and persistent/chronic presentations.

**Fig. 2:**
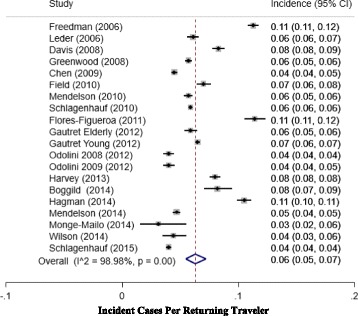
Forest Plot depicting point estimates (95% CI) for the incidence (PM) persistent/chronic diarrhea in Global Returning Travelers

**Fig. 3:**
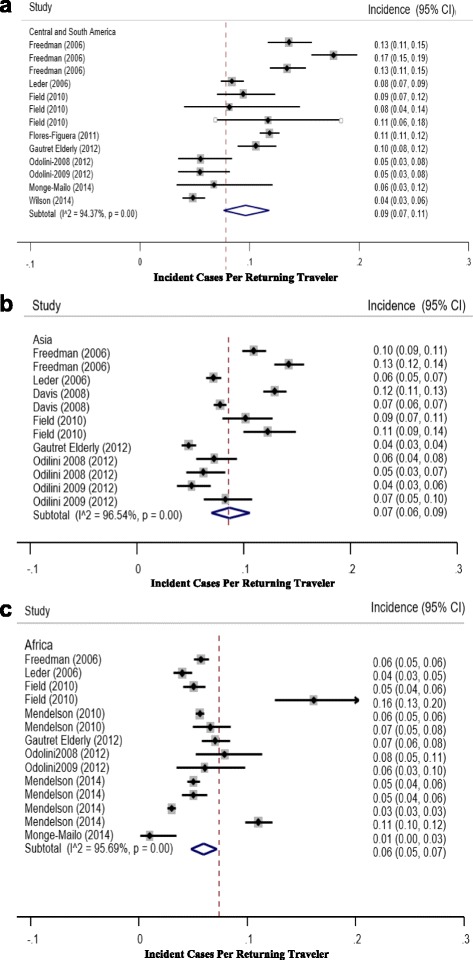
**a-c** Forest Plot depicting point estimates (95% CI) for the incidence (PM) persistent/chronic diarrhea in Returning Travelers Stratified by Continent (Latin America, Africa, and Asia)

The incidence of persistent/chronic diarrhea for travelers from the included studies is shown in Fig. 2. The total incidence for persistent/chronic diarrhea ranged from 0.05 to 0.11. Of note, the highest incidence was identified in Freedman [18] reporting rates from all three regions (0.11 [0.11–0.12]), Hagman [19] reporting global rates (0.11 [0.10–0.11]), and Flores-Figuera [4] reporting rates from Central America (0.11 [0.11–0.12]) [4, 18]. The continental specific [Latin America, Africa, and Asia] number of travelers and incidence (95% CI) was [15816 (0.09 [0.07–0.11]), 42290 (0.06 [0.05–0.07]), and 27433 (0.07 [0.06–0.09]) respectively] depicted in Fig. 3a-c. From the above data we observe significant heterogeneity in incident rates across regions. There was a significant difference (*p* = 0.05) in the incidence of persistent/chronic diarrhea between Africa and Latin America. Persistent/chronic diarrhea ranked fourth as a syndromic diagnosis across all regions. Additionally, within the continental specific incident rates, we observe a trend toward decreasing diarrheal rates in contemporary reporting periods, more pronounced for Latin America and Asia.

**Fig. 4:**
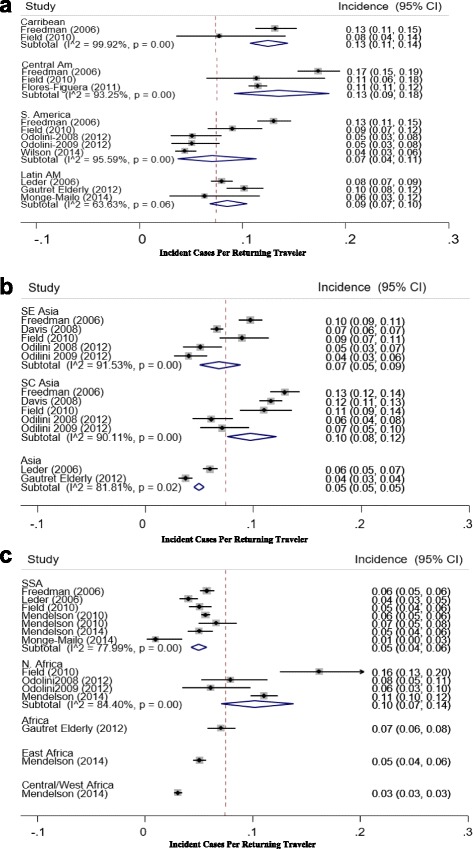
**a-c** Forest Plots depicting point estimates (95% CI) for the incidence (PM) persistent/chronic diarrhea in Returning Travelers Stratified by Continent (Latin America, Africa, and Asia) and Region

Figure 4a-c delineates the persistent/chronic diarrhea incidence by continent and region. For Latin America, we observe a trend towards decreased incidence rates in South America (signifiant relative to the Carribean islands). In Asia, we observe a trend towards decreasing incidence rates observed between SE Asia and SC Asia. Finally, in Africa, we observe a significant difference between incidence rates observed between North Africa and Subsaharan Africa (SSA). Although study numbers are small, we do see significantly lower diarrheal rates between subsaharan Africa and [North Africa, South Central Asia, and Central America].

## Discussion

We estimated 60 cases of persistent/chronic diarrhea per 1000 travelers in over >300,000 global travelers, comparable to a prior report [7]. Although interpretations are limited by the dearth and heterogeneity of studies and variability in outcomes reported, we identified lower published rates of chronic diarrhea from Sub-Saharan Africa relative to North Africa, South Central Asia, and Central America. Of note, persistent/chronic diarrhea was consistently one of the leading syndromic diagnoses across all regions in returning travelers confirming its prominence as a significant public health issue.

The considerable heterogeneity observed in the incidence rates (reflected in the elevated I2 values depicted in all forest plots) best scrutinized within the continental and regional forest plots may be attributed to 1). the historical cohort effect (generally rates have decreased in recent years); 2). varying study designs (passive vice active surveillance); 3) variable enrollment (population sizes and demographics (age, gender, travel origin); 4) heterogeneous travel durations and itineraries; and 5) no control over antecedent travel education and counseling. This heterogeneity contributes to the wide confidence intervals identified upon pooled estimates.

The majority of data identified in our search reporting upon the etiology of travel-related chronic diarrheal infections stem from the GeoSentinel Global Surveillance Network. This surveillance network comprises 57 specialized international travel and tropical medicine clinics in >25 nations on 6 continents contributing sentinel longitudinal surveillance data on all ill travellers, representing the largest repository of travel-related data [20]. This repository provides epidemiologic information on infectious disease burden (and its gradient) in travelers including chronic diarrhea. This network enables communication of novel or emerging disease and outbreaks including diarrheal enteropathogens. As these clinics are referral centers, exploiting passive case ascertainment, accurate epidemiological descriptions (disease and pathogen incidence) may be biased [20] as diagnoses are limited to more chronic, severe or complex diseases leading to underreporting and underrepresentation of the full spectrum and burden of illness [21]. Despite these limitations, the travelers included in this analysis comprise a sentinel cohort facilitating insight into the complex epidemiology of travel-associated chronic diarrhea.

The eligible study reporting enteropathogenic etiologies for persistent/chronic diarrhea in returning travelers [16], coupled to the ineligible studies surveyed, and the unpublished data from the Geosentinel Surveillance Network suggest *Giardiasis* (and other enteropathogenic parasites) comprises an appreciable percentage of infectious mediated etiology [20].

Our study highlights the relative dearth of published data characterizing chronic diarrheal incidence, and enteropathogenic etiologies in infectious-mediated chronic diarrhea in travelers. Although we identified several studies which identified specific enteropathogens associated with chronic diarrhea in returning travelers [11, 17, 22–24], these studies did not sufficiently report denominator, or incidence data. Many studies reported enteropathogenic etiologies for diarrhea across the spectrum of diarrheal acuity without stratifying into chronic (vice acute) categories limiting data interpretation [25, 26].

Despite the lack of etiologic data, a host of case studies affirm a breadth of enteropathogens should be considered [9]. For example, Swaminathan et al. reported travel-associated enteropathogenic etiologies for gastrointestinal disease in a survey of over 25,000 international travelers from 1996 to 2005 exhibiting acute and persistent/chronic diarrhea [26]. Notably, they identified 29% of travelers presenting with infectious gastrointestinal disease (encompassing acute and chronic durations) of which 65% were attributed to parasites, 31% bacterial and 3% viral with significant geographical variation. *Giardia* was the most common pathogen identified (27.9%) followed by *Campylobacter* (13.2%), *E. histolytica* (12.5%), *Shigella* (6.3%), and *Strongyloides* (6.1%) [26]. Soonawala et al. reported on asymptomatic post-travel parasitic carriage in 556 Dutch travelers (median travel 12 weeks, minimum 2 weeks) from 2007 to 2009 to the subtropics. Many of these travelers did report episdoes of acute diarrhea during travel that had resolved by the post-travel evaluation. *Giardia* (4%), *Cryptosporidium spp* (1%), *Schistosoma* spp (6%) (only from travelers to Africa), *and Strongyloides stercoralis* (0.2%) were identified post-travel [25]. Another investigation exploited multiplex PCR for four parasites in fecal specimens acquired from over 2500 Belgium travelers post-travel (regardless of symptoms). They noted the following pathogen distribution: *Giardia lamblia* (6%), *Cryptosporidium* spp (1.3%), *S. stercoralis* (0.8%), *E. histolytica* (0.5%)] [27]. Although these data can not be directly extrapolated to incidence, they do support these parasites as common etiologic agents of aute and chronic TD.

Despite the wealth of data from the GeoSentinel Surveillance Network databases, data on enteropathogen-specific etiology for persistent/chronic diarrhea are lacking. Furthermore, laboratory support was not structured and often limited [18]. Freedman et al. stated that parasitic etiologies accounted for the majority of enteropathogens identified in chronic infectious diarrheal cases presenting to GeoSentinel based clinics from all regions except SE Asia in which bacterial etiologies predominated. We note that many of the eligible studies reported syndromic diagnoses, yet consistently isolated the specific diagnosis of “*Giardiasis*” as an appreciable etiology of all cause travel-related morbidity. For example, Chen [28] reported *Giardiasis* (PM: 36) as the second most common diagnosis in long-term travelers. As travelers evaluated within the GeoSentinel surveillance networks are biased toward persistent/chronic cases, and coupled to the results depicted by Schultsz et al. [16] above, we may infer that *Giardia*is likely a common etiologic agent of infectious persistent/chronic diarrhea [20, 28].

Although not a standardized syndromic diagnosis, many of the studies reported PI-IBS as a major etiology of travel-related morbidity. Recent data culled from the GeoSentinel network (1997–2011) quote a PI-IBS incidence of 2–5% [19, 29] while others have estimated a rate of 5–10% [30, 31]. There was generally a lack of clarity as to whether the PI-IBS diagnosis was rendered in travelers presenting with chronic diarrhea. However, as PI-IBS wasn’t included in the diarrheal estimates, and as PI-IBS cases are biased towards diarrheal presentations [7], we may infer that the incidence of chronic diarrhea in returning travelers is higher than tabulated. Our review provides support for persistent/chronic diarrhea as an important medical issue for the returning traveler and a significant public health issue. We limited our search to publications in the English language. Although we may not have captured literature published in non-English, given that the bulk of the contemporary data derives from the GeoSentinel Global Surveillance Nework whose results are published in the English language, we feel confident we captured the majority of data published.

Despite the limitations cited above, it appears that based on the unpublished and published data supplied by the Geosentinel network, coupled to the eligible and ineligbile studies reported above, that *Giardiasis* and parasites in general comprise an appreciable percentage of infectious mediated persistent/chronic diarrhea in returning travelers [16, 18, 25–27].

To improve our insight into the epidemiologic data on the etiologic agents of travel-associated persistent/chronic diarrhea, systematic investigations utilizing standardized exposure histories, laboratory evaluation and complementary endoscopic evaluation are needed. The use of molecular methods, including multiplex PCR assays on stool specimens, may increase pathogen identification [25, 27]. This would also improve characterization of the non-infectious causes of travel-associated chronic diarrhea, while potentially elucidating the triggers (infectious and non-infectious) and cascades of events precipitating disease unmasking. This information is paramount to developing optimal diagnostic, preventive, and treatment algorithms. The travel clinic is well positioned to conduct these studies contingent upon pursuing active surveillance, and implementing harmonized evaluations across participating clinics.

## Conclusions

Persistent/Chronic diarrhea is a leading syndromic diagnosis globally and across all regions for travel-associated morbidity. The 6% incidence (PM of 60) of persistent/chronic diarrhea observed in over >300,000 global travelers is comparable to prior estimates. We identified lower rates of chronic diarrhea from Sub-Saharan Africa relative to North Africa, South Central Asia, and Central America. Parasites, most notably *Giardia lamblia*, comprise an appreciable percentage of the enteropathogenic etiology of infectious mediated persistent/chronic diarrhea. Our study highlights the relative dearth of published data characterizing chronic diarrheal incidence and pathogen etiology. Ideally, active surveillance investigations desigend to capture incidence data on persistent/chronic diarrhea exploiting the exisitng Travel clinic networks, marshalling standardized exposure histories, and exhaustive and advanced diagnostic methods with delineation of diarrheal duration in returning travelers would fill a significant gap in our understanding of this important public health issue.
